# Role of Institut Hospitalo-Universitaire Méditerranée Infection in the surveillance of resistance to antibiotics and training of students in the Mediterranean basin and in African countries

**DOI:** 10.1016/j.nmni.2018.06.001

**Published:** 2018-06-14

**Authors:** L. Peyclit, A. Chanteloup, L. Hadjadj, J.-M. Rolain

**Affiliations:** Aix-Marseille Université, IRD, APHM, MEPHI, IHU-Méditerranée Infection, Marseille, France

**Keywords:** Africa, Antibiotic resistance, IHU Méditerranée Infection, Mediterranean, Multi-drug resistance

## Abstract

Surveillance of antibiotic resistance has become a public global concern after the rapid worldwide dissemination of several antibiotic resistance genes. Here we report the role of the Institut Hospitalo-Universitaire Méditerranée Infection created in 2011 in the identification and description of multidrug-resistant bacteria thanks to collaborations and training of students from the Mediterranean basin and from African countries. Since the creation of the institute, 95 students and researchers have come from 19 different countries from these areas to characterize 6359 bacterial isolates from 7280 samples from humans (64%), animals (28%) and the environment (8%). Most bacterial isolates studied were Gram-negative bacteria (*n* = 5588; 87.9%), mostly from Algeria (*n* = 4190), Lebanon (*n* = 946), Greece (*n* = 610), Saudi Arabia (*n* = 299) and Senegal (*n* = 278). Antibiotic resistance was diversified with the detection and characterization of extended-spectrum β-lactamases, carbapenemases and resistance to colistin, vancomycin and methicillin. All those studies led to 97 indexed international scientific papers. Over the last 6 years, our institute has created a huge network of collaborations by training students that plays a major role in the surveillance of resistance to antibiotics in these countries.

## Introduction

During the last decade, antibiotic resistance has become one of the major public health priorities in the world [1] because of the emergence of new mechanisms of resistance. Moreover, the massive media coverage has tended to predict of thousands of human deaths every year [2]. However, recent epidemiologic data from our institution demonstrate that the level of antibiotic resistance for the most common bacterial species of clinical interest did not significantly change over the last 15 years in Marseille, France [3], [4]. Similarly, we found a huge disparity between mortality attributable to antibiotic resistance using simple model estimations and empirical data of true deaths in our institution [5]. Data on the level of antibiotic resistance in Europe show disparities between countries and bacterial species for certain antibiotics; for example, resistance to carbapenems is much more frequent in Romania, Italy and Greece [3]. It appears from those studies that a better understanding and surveillance of antibiotic resistance at the local and national levels is critical to manage antibiotic-resistant bacterial infections in the future [5]. However, data on antibiotic resistance and surveillance of the emergence and spread of new mechanisms of resistance in the Mediterranean basin and in African countries were lacking in most of those countries until now.

Here we report the specific and unique role of the Institut Hospitalo-Universitaire Méditerranée Infection (IHU-MI), created in November 2011, in the identification, description and surveillance of multidrug-resistant bacteria thanks to collaborations among and training of students coming from the Mediterranean basin and from African countries in our institute. The majority of students who come to our institute for surveillance and analysis of antibiotic resistance came with their own bacterial isolates from their countries.

## Methods

This study analyses data collected from 2011 (the date of creation of the IHU-MI) through the completion of this article in February 2018. The number of students by level of graduation and by country of origin per year was sorted from our administrative database of students and scientific visitors during the study period from the team dedicated to antibiotic resistance research (JMR team). Students from the Mediterranean basin and Africa were counted from this primary list and were sorted by level of graduation (master's degree, PhD, postdoc and scientific visitors) and by country. Because some students stayed at our institute both for master's and PhD courses, we deduplicated the total count. The number of students present per year in the team was also calculated from this list to show the student kinetics of reception per year.

All students facing antibiotic resistance in their country came with their own isolates to analyse them as a course training. Most of them continued to collaborate with our institute, resulting in real-time surveillance of antibiotic resistance according to their field of research (humans, animals or the environment). Initially there was no rationale for the recruitment and analysis of the samples because no data existed at the beginning of this network. Now, however, the follow-up of antibiotic resistance is mainly focused on the current antibiotic resistance situation. For each epidemiologic study, the number and type of samples and/or bacterial isolates and the country of origin were counted, and data were presented in a single table, with all data provided by country.

Antibiotic resistance for each sample or bacterial isolates was studied using the same procedure. Antibiotic resistance was assessed either directly from samples by PCR or from bacterial isolates by culture and molecular assays. The first step consisted of sample culture and isolation of strains on specific agar media: Columbia agar with 5% sheep's blood, trypticase soy agar or MacConkey (bioMérieux, Marcy l’Etoile, France) with or without addition of antibiotics. All collected strains were subjected to matrix-assisted laser desorption/ionization time-of-flight mass spectrometry (MALDI-TOF MS) for identification [6]. Antibiotic susceptibility testing (AST) was performed using the disc diffusion method on Mueller-Hinton medium agar for phenotypic characterization of the mechanism of resistance. Specific panels of antibiotics were tested according to the bacteria species (e.g. *Enterobacteriaceae,* nonfermentative Gram-negative bacteria, Gram-positive bacteria). Then AST results were interpreted according to European Committee on Antimicrobial Susceptibility Testing guidelines [7]. Genotypic identification of resistance genes were screened by real-time quantitative PCR and confirmed by standard PCR and sequencing when necessary, and sequences were analysed using ARG-ANNOT software [8] to identify the specific antibiotic resistance gene. Multilocus sequence typing was performed to evaluate genetic relatedness of strains. If necessary, a whole genome sequence study was performed to obtain the complete resistome of a strain [9] or to describe the genetic environment of an antibiotic resistance gene [10], [11].

Each student hosted by our institution received specific training for the study of antibiotic resistance (MALDI-TOF MS, AST, molecular training, genomics, bioinformatics) and presented the progress of their work and their results every week so that we could prepare tables and figures to be used for publication. Finally, each student was trained by the senior member of the team (JMR) to write their scientific papers and to create their own bibliography on the topic. Most of them also wrote a review on their topic while writing their PhD thesis. Weekly seminars or bibliographic sessions were also provided each Friday to improve students' knowledge in the field. The number of indexed international scientific papers per type of sample and per country was also calculated on the basis of published and submitted papers on antibiotic resistance during the study period.

## Results

Since the creation of this institute, the JMR team has welcomed a total of 126 students or visiting scientists, including 95 deduplicated students (75.4%) from academic exchanges with 19 countries from the Mediterranean basin, Africa and Middle East. The number of students present in a given year has significantly increased during the study period (ten students in 2011, 15 in 2012, 21 in 2013, 28 in 2014, 27 in 2015, 40 in 2016 and 44 in 2017) to a total of 95 students. Most of the students are from Algeria (52, 55.3%), followed by Lebanon (12, 12.8%), Senegal (7, 7.4%) and Tunisia (5, 5.3%). All these 95 students were from Europe (Spain, 3 students, 3.2%; Italy, 2, 2.1%; Greece, 1, 1.1%), West Africa (Senegal, 7, 7.3%; Benin, Central Africa Republic, Guinea, Ivory Coast, Mali, Nigeria, Togo, 8, 8.4%), North Africa (Algeria, 52, 55.3%; Tunisia, 5, 5.3%; Egypt, 1, 1.1%; Morocco, 1, 1.1%), Middle East (Lebanon, 12, 12.8%; Qatar, Syria, 2, 2.1%) and Madagascar (1, 1.1%).

Each student had a different level of education, including master's degree (*n* = 15), PhD students (*n* = 65), scientific visitors and postdocs (*n* = 22). Overall, the number of PhD students from these countries significantly increased during the study period, from eight in 2011 to 30 in 2017 (2011: 8; 2012: 11; 2013: 16; 2014: 20; 2015: 23; 2016: 28; 2017: 30). The number of postdocs varied from two to four between these different years (2011, 2013, 2015, 2017: 2 students; 2012: 3; 2014, 2016: 4), as did the number of students seeking master's degrees, from one to four (2011, 2012: 1; 2013: 2; 2014, 2015: 3; 2016, 2017: 5). Students trained at the IHU-MI will return to their country of origin and continue to work in this field with our institute, which is now identified as the core laboratory for surveillance of antibiotic resistance and further analysis of new bacterial isolates from those countries.

**Fig. 1:**
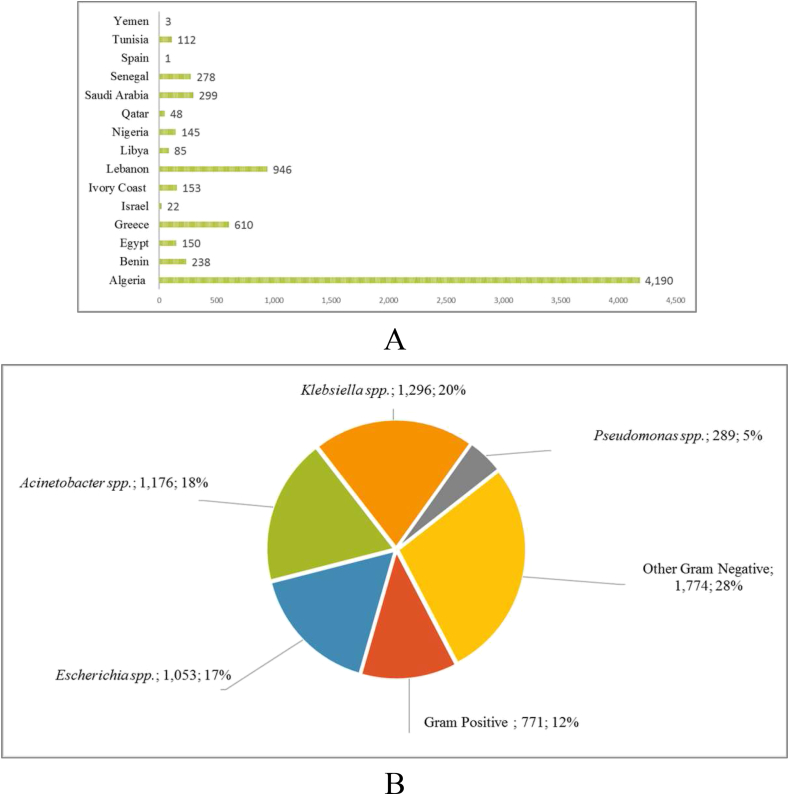
(A) Geographic distribution of samples studied in publications of IHU-MI from 2011 to 2017 (*n* = 7280). (B) Repartition of bacterial species studied from Mediterranean basin or African countries in IHU-MI from 2011 to 2017 (*n* = 6359). IHU-MI, Institut Hospitalo-Universitaire Méditerranée Infection.

A total of 7280 samples from human (*n* = 4657; 64%), animal (*n* = 2058; 28%) or environment (*n* = 565; 8%) from 15 different countries were analysed during the study period (Fig. 1(A)). More than half of those samples came from Algeria (*n* = 4190; 57.6%), followed by Lebanon (*n* = 946; 12.9%), Greece (*n* = 610; 8.4%), Saudi Arabia (*n* = 299; 4.1%) and Senegal (*n* = 278; 3.8%) (Fig. 1(A)). From those 7280 samples, 6359 bacterial isolates were cultured and analysed phenotypically (bacterial identification by MALDI-TOF MS and AST) and genetically (molecular detection of antibiotic resistance genes).

Bacterial genera studied included *Enterobacteriaceae* (1296 *Klebsiella and* 1053 *Escherichia*), followed by *Acinetobacter* (*n* = 1176) or other Gram-negative organisms (*n* = 1774 including bacteria of the genera *Serratia, Salmonella, Enterobacter, Salmonella, Raoultella* and *Shewanella*) and *Pseudomonas* (*n* = 289) (Fig. 1(B)). Gram-positive strains were represented with 771 strains (12%), including bacteria of the genera *Enterococcus, Staphylococcus* or *Streptococcus* (Fig. 1(B)).

All these studies allowed the detection and characterization of specific antibiotic resistance genes in multidrug-resistant bacteria from these genera and led to 97 scientific international indexed publications. Table 1 lists the publications and findings of specific antibiotic resistance determinants by country and bacterial species.

**Table 1:** Samples and publications studied according to countries in Institut Hospitalo-Universitaire Méditerranée Infection from 2011 to 2017

Country	Year	Strain type	Studied strains	No. of samples	No. of positive samples with an AR gene detected	Tested phenotype	Type of antibiotic resistance genes detected (*n*)	Study
Algeria	2008–2011	Clinical isolates	*Klebsiella pneumoniae*	211	194	ESBL Cephalosporinases Penicillinase HLP		[19]
	2008–2012	Clinical isolates	*K. pneumoniae*	221	190	ESBL Cephalosporinases Penicillinase HLP	*bla*_TEM_ (146) *bla*_SHV_ (154) *bla*_CTX-M_ (130)	[20]
	2017	Animal (45) and human (37) isolates	*Salmonella* spp.	92	18	ESBL	*bla*_CTX-M-1_ (12) *bla*_CTX-M-15_ (5) *bla*_TEM_ (8)	[21]
	2011	Clinical isolates	*Escherichia coli*	1	1	Coli R	*mcr-1*	[22]
	2015	Animal isolates	*E. coli* (30) *K. pneumoniae* (17)	47	47	Coli R ESBL	*bla*_CTX-M-15_ (47) *bla*_TEM-1_ (25)	[23]
	2014	Clinical and environmental isolates	*K. pneumoniae* *Enterobacter cloacae* *Acinetobacter baumannii* *Pseudomonas aeruginosa*	89	32	ESBL Carbapenemases	*bla*_OXA-48_ (5) *bla*_NDM-1_ (7) *bla*_OXA-43_ (2) *bla*_TEM_ (6) *bla*_SHV_ + *bla*_CTX-M_ (7)	[24]
	2013–2015	Environmental isolates	*A. baumannii*	1	1	Carbapenemases ESBL Fluoroquinolones	*bla*_NDM-1_ (1)	[25]
	2010–2013	Clinical isolates	*Enterococcus* spp.	85	85	Vanco R	*vanC*	[26]
	2010–2013	Clinical isolates	*A. baumannii*	43	43	Carbapenemases	*bla*_NDM-1_ (7) *bla*_OXA-23_ (28) *bla*_OXA-24_ (1) *bla*_OXA-58_ (6) *bla*_OXA-51_ (43) *bla*_OXA-23_ + *bla*_NDM-1_ (2) *bla*_OXA-58_ + *bla*_NDM-1_ (1)	[27]
	2013–2015	Clinical isolates	*Enterobacteriaceae* (161) *P. aeruginosa* (18) *A. baumannii* (7)	186	36	Carbapenemases ESBL	*bla*_OXA-48_ (2) *bla*_VIM-4_ (2) *bla*_NDM-1_ (2) *bla*_OXA-23_ (5)	[28]
	2011–2013	Environmental isolates	*A. baumannii*	67	61	Carbapenemases	*bla*_OXA-23_ (29) *bla*_NDM-1_ (32)	[29]
	2013–2014	Clinical isolates	*Streptococcus agalactiae*	93	74	MLSB R	*ermB* (18) *ermA* *mef*(*A*) *mef*(*E*)	[30]
	2015	Animal isolates	*E. coli*	1	1	Carbapenemase	*bla*_NDM-5_ (1)	[31]
	2014–2016	Animal isolates	*Enterobacteriaceae*	380	3	Carbapenemases ESBL Cephalosporinases	*bla*_OXA-48_ (3)	[32]
	2012–2014	Environmental isolates	*K. pneumoniae*	44	44	ESBL Cephalosporinases Fluoroquinolones Aminoglycosides	*bla*_CTX-M-15_ (41) *bla*_CTX-M-3_ (3)	[33]
	2014–2015	Animal isolates	Samples	503	389	Carbapenemases ESBL	*bla*_TEM_ (128) *bla*_SHV_ (83) *bla*_CTX-M_ (46) *bla*_OXA-58_ (132)	[34]
	2015	Animal isolates	*Enterobacteriaceae*	32	32	Carbapenemases ESBL	*bla*_OXA-48_ (32) *bla*_CTX-M-15_ (1) *bla*_TEM_ (2)	[35]
	2013–2014	Animal (3) and human (1) isolates	*E. coli*	4	4	Coli R	*mcr-1* (4)	[36]
	2016	Animal isolates	*E. coli*	8	8	Coli R	In progress	Unpublished results
	2017	Animal (4) and environmental (5) isolates	*E. coli* (8) *E. cloacae* (1)	9	9	Coli R	In progress	Unpublished results
	2016	Clinical isolates	*K. pneumoniae*	3	3	Coli R	In progress	Unpublished results
	2015–2016	Environmental isolates	*Staphylococcus aureus*	200	153	Methi R	In progress	Unpublished results
	2011–2013	Clinical isolates	*A. baumannii*	47	47	Carbapenemases Aminoglycosides Fluoroquinolones	*bla*_OXA-23_ (33) *bla*_OXA-24_ (10) *bla*_NDM_ (11) *armA* (4) *aph*(*3′*)*VI* (24) *aadA* (6) *ant*(*2′*)*I* (10) *aac*(*3*)*Ia* (33) Mutation in *gyrA, parC* (45)	[37]
	2013–2014	Clinical isolates	*A. baumannii*	12	12	Coli R Carbapenemases	*bla*_OXA-24_ (4) *bla*_OXA-23_ (6) *bla*_OXA-51_ (12) *bla*_NDM-1_ (2)	[38]
	2016–2017	Animal isolates	Samples	200	4	ESBL	In progress	Unpublished results
	2013	Clinical isolates	*Enterococcus hirae*	1	1			[39]
	2016	Clinical isolates	*K. pneumoniae* (20) *A. baumannii* (12) *P. aeruginosa* (9) *E. coli* (27)	68	68	Carbapenemases ESBL	In progress	Unpublished results
	2011–2012	Clinical isolates	*A. baumannii*	30	24	Carbapenemases	*bla*_OXA-23_ (22) *bla*_OXA-58_ (1) *bla*_OXA-23_ + *bla*_OXA-58_ (1)	[40]
	2011	Clinical isolates	*P. aeruginosa*	17	17	Carbapenemases	*bla*_VIM-2_ (14) Mutation in *oprD* (3)	[41]
	2012–2013	Clinical isolates	*E. coli*	105	3	Carbapenemases ESBL Aminoglycosides Fluoroquinolones	*bla*_NDM-5_ (3) *bla*_TEM_ (3) *bla*_CTX-M_ (3) *aadA* (3)	[42]
	2010–2011	Clinical isolates	*E. coli* (3) *K. pneumoniae* (24) *E. cloacae* (11) *Serratia marcescens* (4)	42	39	ESBL	*bla*_CTX-M_ (10) *bla*_TEM_ (14) *bla*_SHV_ (15)	[43]
	2010–2011	Clinical isolates	*K. pneumoniae*	100	100	ESBL Aminoglycosides Fluoroquinolones	*bla*_CTX-M_ (76) *bla*_TEM_ (74) *bla*_SHV_ (73) *armA* (23) *aadA* (35) *aac*(*6′*)*Ib* (50) *qnrB* (22)	[44]
	2013	Animal isolates	*Acinetobacter* spp.	33	4	Carbapenemases	*bla*_OXA-23_ + *bla*_OXA-58_ + *bla*_OXA-51_ (1) *bla*_OXA-58_ + *bla*_OXA-51_ (1) *bla*_OXA-58_ (2)	[45]
	2014–2015	Clinical isolates	*K. pneumoniae*	7	7	ESBL Carbapenemases	*bla*_OXA-48_ + *bla*_CTX-M-15_ + *bla*_SHV-1_ + *bla*_TEM-1D_ (7)	[46]
	2015	Environmental isolates	*Enterobacteriaceae*	12	9	Carbapenemases ESBL	*bla*_CTX-M-15_ (9) *bla*_OXA-48_ (1) *bla*_TEM_ (1)	[47]
	2015	Animal isolates	*Pseudomonas putida*	1	1	Carbapenemases ESBL	*bla*_VIM-2_	[48]
	2017	Clinical isolates	*K. pneumoniae*	1	1	Carbapenemases ESBL	*bla*_OXA-48_ (1) *bla*_SHV-27_ (1)	[49]
	2010–2011	Clinical isolates	*A. baumannii*	71	71	Carbapenemases	*bla*_OXA-23_ (31) *bla*_OXA-24_ (5) *bla*_OXA-51_ (71)	[50]
	2010–2011	Clinical isolates	*A. baumannii*	71	71	Carbapenemases ESBL Aminoglycosides Fluoroquinolones	*bla*_TEM_ (53) *ampC* (69) *aph*(*3′*)*VI* (36) *aadA* (45) *ant*(*2′*)*I* (10) *aac*(*3*)*Ia* (64) *aac*(*6′*)*Ib* (3) Mutation *gyrA, parC* (67)	[51]
	2013	Clinical isolates	*K. pneumoniae*	1	1	Carbapenemases ESBL Aminoglycosides Fluoroquinolones	*bla*_KPC-3_ *bla*_TEM_ *bla*_SHV_ *aac*(*6′*)*Ib* *aadA*	[52]
	2015	Environmental isolates	*E. coli* (12) *K. pneumoniae* (3) *Raoultella ornithinolytica* (3) *Citrobacter freundii* (1) *Citrobacter braakii* (1)	20	20	ESBL Carbapenemases	*bla*_OXA-48_ (17) *bla*_OXA-24_4 (3) *bla*_TEM-1_ (9) *bla*_CTX-M-15_ + *bla*_TEM-1_ (3)	[13]
	2016	Environmental isolates	*K. pneumoniae*	87	3	ESBL Carbapenemases	*bla*_OXA-48_ (3) *bla*_TEM-1_ (1)	[16]
	2014	Animal isolates	*E. coli*	20	20	Carbapenemases ESBL Aminoglycosides	*bla*_TEM-1_ (20) *bla*_CTX-M-1_ (2) *bla*_SHV_-12 (14) *CMY-2* (4) *aadA* (20)	[12]
	2015	Environmental isolates	*Shewanella xiamenensis*	4	4	Carbapenemases	*bla*_OXA-48_ (1) *bla*_OXA-19_9 (1) *bla*_OXA-18_1 (2)	[11]
	2016	Animal isolates	*Enterobacteriaceae*	86	1	Coli R	*mcr-1* *bla*_CTX-M-15_ *bla*_TEM-1_ *qnrB19*	[17]
	2014	Clinical isolates	*S. aureus*	250	171	Methi R	In progress	Unpublished results
	2005–2007	Clinical isolates	*S. aureus*	64	64	Methi R	*mecA* (64)	[53]
	2011	Clinical isolates	*Proteus mirablis* *Morganella* spp. *Providencia* spp.	106	72	ESBL Carbapenemases Aminoglycosides Fluoroquinolones	In progress	Unpublished results
	2014–2015	Clinical (60) and environmental (39) isolates	*Enterobacteriaceae* *Acinetobacter* spp.	99	10	Carbapenemases ESBL Cephalosporinases Aminoglycosides	*bla*_NDM-1_ (5) *bla*_OXA-23_ (3) *bla*_OXA-48_ (2) *bla*_SHV-148_ + *bla*_TEM-163_ (2) *aph*(*3′*)*VI–ant*(*2″*)*I* (2) *aac*(*3*)*Ia aadA* (3) AME-encoding genes (5)	[54]
	2009–2012	Clinical isolates	*P. aeruginosa*	89	39	Carbapenemases Aminoglycosides	*bla*_VIM-2_ (2) *aadA* (10) *aac*(*3*)*Ia* (3)	[55]
	2008–2012	Clinical isolates	*Acinetobacter* spp.	113	113	Carbapenemases ESBL Aminoglycosides Fluoroquinolones	*bla*_OXA-23_ (40) *bla*_OXA-24_ (17) *bla*_NDM_ (5) *aph*(*3′*)*VI* (70) *aadA* (57) *ant*(*2′*)*I* (60) *aac*(*3*)*Ia* (77) *aac*(*6′*)*Ib* (1)	[56]
	2012	Clinical isolates	*A. baumannii*	123	77	Carbapenemases ESBL	*bla*_OXA-23_ (40) *bla*_OXA-24_ (3) *bla*_OXA-23_ + *bla*_OXA-24_ (3)	[57]
Benin	2015	Clinical isolates	*Staphylococcus saprophyticus* (31) *S. aureus* (21) *Staphylococcus sciuri* (17) *Staphylococcus conhii* (5) *Staphylococcus haemolyticus* (2) *Staphylococcus xylosus* (1) *Staphylococcus hominis* (1)	78	21	Methi R	*mecA* (19)	[58]
	2015	Clinical isolates	*Enterobacteriaceae*	157	103	ESBL Carbapenemases	In progress	Unpublished results
	2016	Clinical isolates	*P. aeruginosa*	3	3	Carbapenemases	In progress	Unpublished results
Egypt	2012–2013	Clinical isolates	*A. baumannii*	150	150	Carbapenemases Aminoglycosides	*bla*_NDM-1_ (59) *bla*_OXA-23_ (115) *armA* (141) *bla*_OXA-51_ (150) *bla*_NDM-1_ + *bla*_OXA-23_ (53) *armA* + *bla*_NDM-1_ + *bla*_OXA-23_ (52)	[59]
Greece	2013–2017	Clinical isolates	*P. mirablis* (4) *P. putida* (1) *C. freundii* (1) *Enterobacter aerogenes* (2) *Providencia stuartii* (8) *P. aeruginosa* (79) *A. baumannii* (158) *E. cloacae* (10) *E. coli* (33) *K. pneumoniae* (314)	610	610	Carbapenemases ESBL Coli R Fluoroquinolones	In progress	Unpublished results
Israel	2011	Clinical isolates	*Providencia rettgeri*	1	1	Carbapenemase	*bla*_NDM-1_ (1)	[60]
	2008–2011	Clinical isolates	*K. pneumoniae*	15	15	Carbapenemases Coli R Aminoglycosides	In progress	Unpublished results
	2010–2011	Clinical isolates	*K. pneumoniae* (1) *E. coli* (1) *P. mirabilis* (1) *P. rettgeri* (1) *Morganella morganii* (1)	5	5	Carbapenemases	*bla*_NDM-1_ (5)	[61]
	2014	Clinical isolates	*M. morganii*	1	1	Carbapenemases	*bla*_NDM-1_ (1)	[62]
Ivory Coast	2012–2015	Clinical isolates	*Enterobacteriaceae*	153	153	ESBL Aminoglycosides Fluoroquinolones	In progress	Unpublished results
Lebanon	2013	Clinical isolates	*P. aeruginosa*	35	35	Carbapenemases Cephalosporinases	*bla*_VIM-2_ (16) *bla*_IMP-15_ (2) *ampC* (8)	[14]
	2013	Animal isolates	*E. coli*	1	1	ESBL Carbapenemases	*bla*_OXA-48_ (1) *bla*_CTX-M-1_4 (1) *bla*_TEM-1_ (1)	[63]
	2013	Animal isolates	*P. aeruginosa* (4) *A. baumannii* (5)	9	9	Carbapenemases	*bla*_OXA-23_ (4) *bla*_OXA-58_ (1) *bla*_VIM-2_ (4)	[64]
	2015	Clinical isolates	*R. ornithinolytica*	1	1	Carbapenemases Cephalosporinase BLSE MLSB R Chloramphenicol Fluoroquinolones	*bla*_OXA-48_ *ampC* *ampH* *bla*_TEM-166_ *macA* *macB* *cmr* *cat* *gyrA* mutated	[10]
	2015	Animal isolates	*E. coli*	1	1	ESBL Coli R	*bla*_TEM-135_-like (1) *mcr-1* (1)	[18]
	2015	Animal isolates	*E. cloacae*	1	1	Cephalosporinase Coli R	In progress	Unpublished results
	2015	Animal isolates	*E. coli* (217) *K. pneumoniae* (8) *Escherichia fergusonii* (1) *A. baumannii* (1) *P. mirabilis* (3) *E. cloacae* (2)	235	235	ESBL Cephalosporinases	In progress	Unpublished results
	2017	Animal isolates	*E. coli* (105) *E. fergusonii* (2) *K. pneumoniae* (4)	111	111	ESBL Cephalosporinase Coli R	In progress	Unpublished results
	2017	Animal (346) Environmental (53) Human (11) isolates	*E. coli* (341) *K. pneumoniae* (31) *Enterobacter asburiae* (1) *Stenotrophomonas maltophilia* (4) *Serratia rubidae* (1) *A. baumannii* (4) *Acinetobacter genomospecies* (4) *Pseudomonas* spp. (8) *Ochrobactrum* spp. (1) *E. cloacae* (4)	399	399	ESBL Cephalosporinase Coli R	In progress	Unpublished results
	2016–2017	Clinical isolates	*Enterobacter faecium*	4	4	Glycopeptides	In progress	Unpublished results
	2016	Clinical isolates	*A. baumannii*	31	31	ESBL Carbapenemases	In progress	Unpublished results
	2016	Clinical isolates	*Campylobacter jejuni*	1	1	ESBL Carbapenemases	In progress	Unpublished results
	2010–2016	Clinical isolates	*E. coli*	43	43	ESBL	In progress	Unpublished results
	2015	Clinical isolates	*K. pneumoniae*	3	3	ESBL Coli R	*bla*_CTX-M-15_ + *bla*_TEM-12_ + *bla*_SHV-5_ (2) *bla*_SHV-5_ (1) Mutation *mgrB* (2) *phoQ* (1) *pmrA* (1)	[65]
	2016–2017	Clinical isolates	*Enterobacteriaceae* (8) *P. aeruginosa* (1)	9	9	ESBL Carbapenemases Coli R	In progress	Unpublished results
	2012	Clinical isolates	*A. baumannii*	4	4	Carbapenemases	*bla*_NDM-1_ (4) *bla*_OXA-94_ (4)	[15]
	2016	Clinical isolates	*Neisseria meningitidis*	58	1		In progress	Unpublished results
Libya	2013–2014	Clinical isolates	*P. aeruginosa* (24) *A. baumannii* (25)	49	43	Carbapenemases	*bla*_OXA-24_ (3) *bla*_OXA-23_ (19) *bla*_VIM-2_ (19)	[66]
	2015	Clinical isolates	*A. baumannii*	36	36	Carbapenemases	*bla*_OXA-23_ (29) *bla*_NDM-1_ (8) *bla*_NDM_ (36)	[67]
Nigeria	2012	Clinical isolates	*A. baumannii*	3	3	Carbapenemases	*bla*_OXA-23_ (3)	[68]
	2012–2013	Clinical isolates	*Klebsiella* spp.	139	1	Coli R	*mgrB* (1)	[69]
	2012	Animal (2) and human (1) isolates	*E. coli*	3	1	Coli R	*mcr-1* (1)	[70]
Qatar	2011–2012	Clinical isolates	*A. baumannii*	48	48	Carbapenemases	*bla*_OXA-23_ (48)	[68]
Saudi Arabia	2013–2014	Clinical isolates	*E. coli* (10) *K. pneumoniae* (1)	11	11	Coli R ESBL	*mcr-1* (11) *bla*_TEM-1_ (10) *bla*_SHV_-1 (1) *bla*_CTX-M-15_ (1)	[71]
	2014	Clinical isolates	*A. baumannii*	42	28	Carbapenemases ESBL Aminoglycosides	*oxa-72* (1) *bla*_NDM-5_ (1) *bla*_NDM-1_ (1) *bla*_OXA-48_ (1) *bla*_OXA-58_ (22) *bla*_OXA-51_ + *bla*_OXA-72_ (1) *bla*_NDM-5_ + *bla*_CTX-M-15_ + *bla*_TEM-1_ + *aadA2* (1) *bla*_NDM-5_ + *bla*_TEM-1_ + *aadA2* (1)	[72]
	2013–2014	Clinical isolates	*E. coli* (23) *K. pneumoniae* (5)	28	28	ESBL	*bla*_CTX-M_ (27) *bla*_TEM_ (19) *bla*_SHV_ (4)	[73]
	2013–2014	Clinical isolates						[74]
	2013–2014	Clinical isolates	Samples	218	73	ESBL	*bla*_CTX-M_ (73)	[75]
Senegal	2011	Clinical isolates	*A. baumannii*	5	3	Carbapenemases	*bla*_OXA-23_ (3)	[76]
	2014	Clinical isolates	*M. morganii*	112	112	ESBL	*bla*_CTX_ (112) *bla*_TEM_ (86) *bla*_SHV_ (63)	[77]
	2015–2017	Clinical isolates	*Enterobacteriaceae*	161	120	Carbapenemases ESBL Aminoglycosides Fluoroquinolones Coli R	In progress	Unpublished results
Spain	2015	Clinical isolates	*Acinetobacter nosocomialis*	1	1	Coli R		[78]
Tunisia	2013–2016	Clinical isolates	*A. baumannii*	25	25	Carbapenemases	*bla*_OXA-51_ + *bla*_OXA-23_ (25) *bla*_OXA-58_ (1)	[79]
	2015	Clinical isolates	*E. coli* (51) *K. pneumoniae* (36)	87	68	Carbapenemases ESBL	*bla*_CTX_ (47) *bla*_TEM-1_ (31) *bla*_SHV_ (18) *bla*_OXA_ (10)	[80]
Yemen	2013	Clinical isolates	*A. baumannii*	3	3	Carbapenemases Aminoglycosides Fluoroquinolones	*bla*_OXA-23_ (3) *armA* (3) *aac*(*6′*)*Ib* (1) Mutated *gyrA* (3)	[81]
			Total studied samples	7280			Total no. of publications	97
			Total studied strains	6359				

‘Samples’ indicates that no strains were isolated but samples were directly tested by PCR.

Coli R, colistin resistance; ESBL, extended-spectrum β-lactamase; HLP, high-level penicillinase; Methi

R, methicillin resistance; MLSB, macrolide–lincosamide–streptogramin B phenotype; Vanco R, vancomycin resistance.

**Fig. 2:**
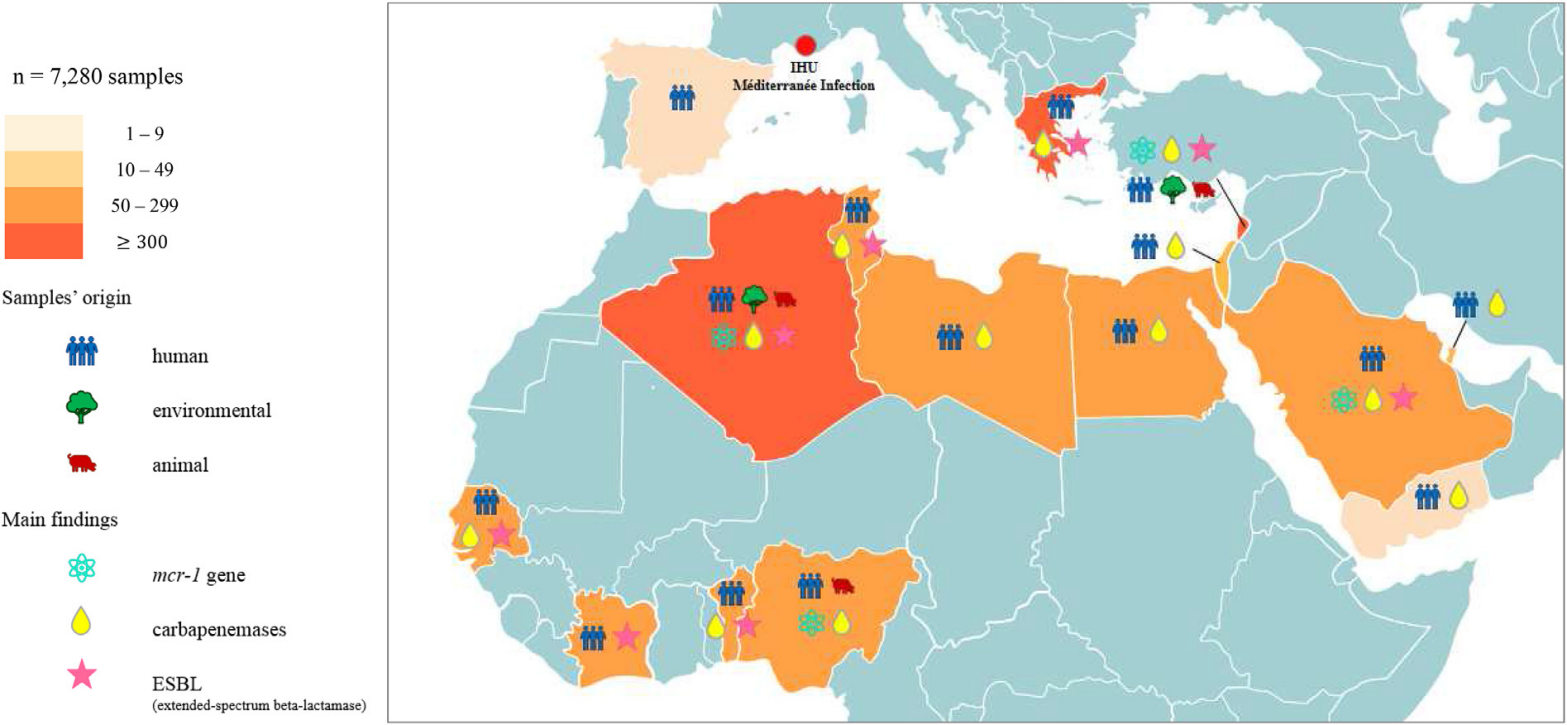
Global distribution of samples in Mediterranean basin and African countries.

The main antibiotic resistance determinants detected and characterized were extended-spectrum β-lactamases (ESBLs) and carbapenemases including *bla*_CTX-M_, *bla*_SHV_, *bla*_TEM_ [12], *bla*_OXA-48_ [13], *bla*_NDM_ [82] and *bla*_VIM_ [14] genes. Genes encoding for resistance to aminoglycosides were also reported, including, for example, *armA* or *aac*(*6′*)*-Ib* in *Acinetobacter baumannii* [81]. Resistance to colistin mediated by the newly plasmid-mediated *mcr-1* gene in human and animal isolates has been tested to date in 21 studies from eight countries (Algeria, Greece, Israel, Lebanon, Nigeria, Saudi Arabia, Senegal and Spain), leading to ten scientific publications (Table 1). An overview of the global distribution of the main findings of antibiotic resistance determinants in the 7280 samples studied per country and type of samples is provided in the map in Fig. 2.

## Discussion

Here we show the unique role of IHU-MI in training about 100 students working in the field of antibiotic resistance from the Mediterranean basin and Africa over the last 6 years. This has led to the description and surveillance of new mechanisms of resistance to antibiotics in 15 various countries reported in 97 scientific publications, including 24 different peer-reviewed journals. The majority of the publications have reported the first detection of antibiotic resistance genes, mainly ESBLs [47], carbapenemases [15], [16], [31] and the *mcr-1* plasmid-mediated colistin resistance gene [17], [18], [22] in these countries in both humans and animals. One of the main contributions in the field is the description of a strong link between antibiotic consumption in animals and emergence and spread of antibiotic resistance genes in animals as well as the transfer to humans [83].

Antibiotics are widely used in agricultural settings in these countries, without clear control policies; this situation has affected human health and is implicated in the evolution of new mechanisms of resistance [84]. Epidemiologic descriptions are essential, and our results confirmed that surveillance should continue in Africa and in the Mediterranean basin to monitor and control the emergence and spread of antibiotic resistance genes. Thanks to these students and their training at the IHU-MI, the institute has created a unique collaborative network for surveillance and study of antibiotic resistance in Africa and in the Mediterranean basin because most of these students returned to their country of origin and created microbiology laboratories to study and survey antibiotic resistance in collaboration with the IHU-MI institute. Further engagements with key individuals are ongoing to create new partnerships to study antibiotic resistance in humans and animals in Italy, Morocco, Turkey and in the Balkans, and should be reinforced in Egypt and Libya as well as the Middle East, although the political situation is currently complex. The current migrant crisis in Europe should prompt us to survey antibiotic resistance in humans and animals from these countries, including Syria and Iraq, to avoid the possible spreading of specific clones, as previously reported in Greece [85], [86] and Israel [87] for *Klebsiella pneumoniae* carbapenemase producers.

Because of its special location as a seaport in the Mediterranean basin, Marseille has historically always been a critical place for the entrance of infectious diseases such as plague or cholera [88]. Because antibiotic-resistant bacteria and antibiotic resistance genes that could spread in the Mediterranean basin do not have borders, the IHU-MI in Marseille plays a critical role in the surveillance of resistance in these areas as well as in African countries that historically have links to France. Thus, over the last 6 years, the institute has become a reference centre for the surveillance of antibiotic resistance and the training of students from countries in the Mediterranean basin and Africa. Such a collaborative network will expand in the future, permitting real-time surveillance of antibiotic resistance determinants that may emerge and spread in these areas [89].
